# Gender Differences in Digital Learning During COVID-19: Competence Beliefs, Intrinsic Value, Learning Engagement, and Perceived Teacher Support

**DOI:** 10.3389/fpsyg.2021.637776

**Published:** 2021-03-30

**Authors:** Selma Korlat, Marlene Kollmayer, Julia Holzer, Marko Lüftenegger, Elisabeth Rosa Pelikan, Barbara Schober, Christiane Spiel

**Affiliations:** ^1^Department of Developmental and Educational Psychology, University of Vienna, Vienna, Austria; ^2^Department for Teacher Education, Centre for Teacher Education, University of Vienna, Vienna, Austria

**Keywords:** gender differences, gender role self-concept, digital learning, COVID-19, adolescents

## Abstract

The spread of the COVID-19 pandemic quickly necessitated digital learning, which bore challenges for all pupils but especially for groups disadvantaged in a virtual classroom. As some studies indicate persistent differences between boys and girls in use of technologies and related skills, the aim of this study was to investigate gender differences in the digital learning environment students faced in spring 2020. Previous studies investigating gender differences in digital learning largely used biological sex as the only indicator of gender. This study includes both biological sex and gender role self-concept in order to investigate the role of gender in different components of this stereotyped domain in a more differentiated way. A total of 19,190 Austrian secondary school students (61.9% girls, *M*_age_ = 14.55, SD_age_ = 2.49, age range 10–21) participated in an online study in April 2020 and answered questions regarding their competence beliefs, intrinsic value, engagement, and perceived teacher support in digital learning during the pandemic-induced school closures. Results showed higher perceived teacher support, intrinsic value, and learning engagement among girls than boys, while no significant sex differences were found in competence beliefs regarding digital learning. Furthermore, our results indicated clear benefits of an androgynous gender role self-concept for all studied components of digital learning. Implications of the findings for theory and practice are discussed.

## Introduction

Due to the spread of COVID-19, countries worldwide implemented unprecedented measures in various sectors of society to contain the pandemic (OECD, 2020). This situation affected the education sector as well, causing the largest disruption of education systems in history (UN, 2020). As of March 2020, a majority of countries had announced temporary school closures, preventing around 1.6 billion children and young people from physically attending school (UNICEF, 2020). As a response, most schools switched to digital learning, creating a unique situation for all actors in the education field (UN, 2020). While various European Union bodies and international organizations had long called for technology adoption in education systems (OECD, 2001; European Commission, 2018), most European school systems had continued to employ face-to-face teaching as their main *modus operandi* before COVID-19 (Wahlmüller-Schiller, 2017; Schrenk, 2020). The urgent imperative to move online following the outbreak of the virus forced digital learning upon unprepared school systems (Hodges et al., 2020), putting at risk all pupils but especially groups that might be particularly disadvantaged in the virtual classroom. UNESCO and scholars have called for recognizing the gender dimension of school closures due to COVID-19, especially in light of the gender digital gap (IIEP-UNESCO, 2020; Nefesh-Clarke et al., 2020). As there are studies indicating that gender differences persist in use of technologies and related skills (Kayany and Yelsma, 2000; Colley and Comber, 2003; Li and Kirkup, 2007; Drabowicz, 2014), it is critical to investigate gender differences in important components of digital learning—a stereotyped domain that became a necessity in schoolchildren lives during COVID pandemic.

### Gender Differences in Stereotyped Domains

In line with gender stereotypes associating technical and math-intensive fields with masculine qualities (Charles and Bradley, 2009), computers and technology use have been perceived as masculine and therefore more suitable for boys than girls (Cooper, 2006; Adamus et al., 2009). The “digital gender gap” begins in early childhood, as parents and teachers act in accordance with the perception that computers are a male domain (Young, 2000). From earliest infancy, boys' activities and toys tend to relate to technology and action, whereas girls' activities and toys relate to nurturance and beauty (Blakemore and Centers, 2005; Kollmayer et al., 2018). Similarly, it has been shown that parents provide boys with more opportunities to do computing and sports, whereas girls are enabled more to read and to interact socially with their peers (Eccles et al., 1993). Hence, these gendered experiences can undermine girls' confidence in their abilities and interest in computing-related subjects (Eccles, 2009). Accordingly, by the time of adolescence, boys report higher frequency of computer use and greater self-confidence in dealing with computers (Colley and Comber, 2003; Mucherah, 2003), display greater digital skills (Kayany and Yelsma, 2000; Li and Kirkup, 2007), and in general are more attracted to computers than girls (Mumtaz, 2001; Volman and van Eck, 2001; Colley and Comber, 2003). Similarly, it has been found that boys describe themselves in relation to computers (e.g., “computer freak” or “I like computers”) significantly more often than girls (Korlat et al., 2021). Newer studies support the finding that computer use for both education and entertainment purposes is more frequent among boys (Drabowicz, 2014). Girls, on the other hand, seem to use computers and the internet more for communication and social networking (McSporran and Young, 2001). As boys' and girls' motivational beliefs and behaviors are shaped by their experiences and are a result of gendered socialization processes (Eccles, 1994; Meece et al., 2006), the gender digital gap corresponds to societal gender stereotypes that portray boys as autonomous, independent, and good at technology-related domains and girls as gentle, sociable, and good at nurturing domains. Not only can this influence girls' use of computers but it may also have far-reaching consequences for girls' education and career selection (Van Grootel et al., 2018), thus contributing to the “leaky pipeline” in science, technology, engineering, and mathematics (STEM) as well as the continued gendered division of labor (Wood and Eagly, 2012).

Eccles-Parsons et al. (1983) developed a model to explain gender differences in adolescents' achievement choices and lower proportion of girls and women in advanced high school math courses or math and science careers (Wigfield and Eccles, 2020). According to their *expectancy-value model* (Eccles-Parsons et al., 1983; Wigfield and Eccles, 2000), students are more likely to engage in academic activities within the range of their perceived ability to successfully perform them (competence beliefs) and which they consider valuable in terms of the enjoyment they will get from the task (subjective task value). Previous studies found both competence beliefs and values as important predictors for engagement and achievement in gender-stereotyped STEM subjects (Chow et al., 2012; Watt et al., 2012). However, there is evidence of gender differences in both ability-related and subjective task value-related beliefs in stereotyped domains (Eccles, 2009). For instance, girls reported lower competence beliefs in sports but higher competence beliefs in language arts compared to boys (Jacobs et al., 2002; Lupart et al., 2004). Similarly, some studies showed lower competence beliefs in mathematics for girls compared to boys (e.g., Lupart et al., 2004; Herbert and Stipek, 2005). In the similar vein, girls reported liking math and physics less than boys and rated math as less useful than boys (Eccles and Harold, 1991; Eccles, 2011). Persistent differences favoring boys has been found also for engagement in STEM fields (e.g., Moss-Racusin et al., 2018). To explain these sex differences in stereotyped domains, Eccles-Parsons et al. (1983) theorized that men and women acquire different patterns of competence beliefs and values, and consequently different levels of engagement across various activities, which are aligned with their gender role due to divergent gender-role socialization. In a longitudinal study of adolescent life transitions, they found that girls placed more value than boys on the importance of making occupational sacrifices for one's family, whereas boys placed more value on seeking out challenging tasks and doing work that involves the use of math and computers (Eccles, 2007). The authors argued that, when investigating precursors of competence beliefs and values, focus should not be merely on sex differences but on gender roles and level of fitness of the task with one's gender role self-concept developed through the socialization process (see Eccles, 2009).

### The Role of Gender in Digital Learning

As girls seem to face specific barriers and difficulties in their experiences with computers and information and communication technologies (ICT) in general, concerns about equity in digital learning have been raised (Yates, 2001; Price, 2006). Specifically, it has been suggested that boys may have an advantage over girls in the online classroom solely based on their higher perceived ability, comfort, and engagement with computers (Ashong and Commander, 2012). However, the results of studies investigating sex differences in this context are heterogeneous. While boys have a clear advantage over girls in confidence in their ICT abilities (Mumtaz, 2001; Durndell and Haag, 2002; Broos, 2005; Broos and Roe, 2006; Meelissen and Drent, 2007)—and this pattern seems to be quite consistent from elementary school to university (see Vekiri and Chronaki, 2008, for a review)—a more recent meta-analysis with university students revealed higher competence beliefs regarding learning in digital setting in young women compared to young men (Perkowski, 2013). This might be due to higher academic competence beliefs in girls and women (Britner and Pajares, 2001) that annuls the negative stereotyped effects in this digital context. When it comes to values toward ICT and digital learning, some research has shown that girls tend to have less positive beliefs about the value of ICT and about their own ICT skills compared to boys (Volman and van Eck, 2001), have less positive perceptions of digital learning (Ong and Lai, 2006), and have lower satisfaction with digital learning than male students (Lu and Chiou, 2010). On the other hand, there are studies suggesting that there are no differences between boys and girls in attitudes toward digital learning (Cuadrado-García et al., 2010; Hung et al., 2010) or in average ICT participation and motivation (Cuadrado-García et al., 2010). Other studies indicate advantages for girls when it comes to learning motivation in digital contexts (e.g., McSporran and Young, 2001; Price, 2006). In general, some authors argue that sex differences in digital competence, attitudes, and motivation are becoming less prevalent, indicating a narrowing of the gender digital gap (e.g., Vekiri, 2013).

However, as ICT is perceived as a stereotypically masculine field, it seems plausible that gender differences in digital learning map onto students' gender role self-concepts rather than their biological sex. The recognition that individuals can describe themselves in terms of both stereotypically feminine and stereotypically masculine attributes regardless of their biological sex has led to an increased focus on gender role self-concept and its relationship with gendered domains (e.g., Athenstaedt, 2002; Kessels and Steinmayr, 2013; Wolter and Hannover, 2016). Previous studies have shown that adolescents who describe themselves using masculine qualities (e.g., independent, competitive, and brave) have higher perceived mathematics-related competence (Wolter and Hannover, 2016) and performance (Signorella and Jamison, 1986), whereas adolescents who describe themselves with feminine traits (e.g., gentle, kind, and sensitive) have better reading performance and motivation in reading—a stereotypically feminine domain (McGeown et al., 2012; Wolter and Hannover, 2016). Furthermore, it has been found that individuals high on both masculinity and femininity—androgynous individuals—are more flexible and adaptable to different situations, as they possess a broader repertoire of traits and behaviors (e.g., Bem, 1981; Pauletti et al., 2017). Conversely, individuals scoring low on both dimensions—undifferentiated individuals—exhibit the lowest levels of adaptability and functioning (Markstrom-Adams, 1989; Pauletti et al., 2017). Despite the significant role of gender role self-concept for adolescents' competence and value-related beliefs and engagement regarding gendered domains, studies investigating gender differences in digital learning have so far concentrated on biological sex only, neglecting the role of gender role self-concept. Moreover, all previous studies on sex differences in digital learning were conducted pre-pandemic when pupils were not necessarily continuously exposed to it, especially not in the mandatory and exclusive form of learning as they are during the pandemic lockdowns. Therefore, the goal of this study was to include both biological sex and gender role self-concept in order to investigate gender differences in digital learning context during pandemic-induced school closures.

### Perceived Support

Except for personal characteristics such as one's gender identity, learning achievement is influenced by a broad array of social factors, which include socializers' (especially parents' and teachers') beliefs and behaviors (Eccles, 2009). Although parental beliefs are significant predictors of youths' motivational beliefs and behavior (Eccles et al., 1993; Simpkins et al., 2012), studies have indicated that support from teachers most accurately predicts school-related variables (Ryan et al., 1994; Demaray et al., 2005). Indeed, it has been shown that teachers' support is positively related to competence beliefs regarding academic skills (Patrick et al., 2007), intrinsic motivation (Ryan et al., 1994), and achievement. Some studies report higher levels of perceived teacher affective support among girls (e.g., Reddy et al., 2003), whereas other studies indicate that boys and girls perceive similar levels of teacher support (Malecki and Demaray, 2003; De Wit et al., 2010). Teachers' ability expectations are influenced by their domain-specific gender stereotypes (Chalabaev et al., 2009), which can influence boys' and girls' competence beliefs about ICT through differences in communication patterns or pedagogical practices (Crombie et al., 2002). However, research investigating teacher support for boys' and girls' digital learning are scarce. Vekiri (2010), for instance, found no differences between boys and girls in perceived teacher expectations and support but a stronger association between teacher support and girls' competence beliefs. Nevertheless, perceived teacher support is even more important in digital learning setting, particularly in a situation such as the COVID-19 pandemic, in which students' motivation may begin to degrade if they lack the motivational regulation needed to succeed in this learning setting (Fryer and Bovee, 2016).

### Present Study

The primary goal of goal of the current study is to test gender differences within the expectancy-value model (Eccles-Parsons et al., 1983; Eccles, 2005) in components of digital learning relevant for learning process during pandemic-induced school closures. As the model posits different patterns of competence and value-related beliefs and engagement across various activities associated with gender roles in boys and girls (Eccles, 2009), this study encompasses both biological sex as well as gender role self-concept in investigating gender differences in digital learning during COVID-19 pandemic. Although Eccles and colleagues have suggested gender roles as a factor influencing attainment value as an aspect of subjective task value that is most related to broader identity issues (cf., Eccles, 2009), a recent study showed that components of students' task values (intrinsic, attainment, and utility values) relate to one another, with the correlations being quite high, in the context of stereotyped STEM classes (Perez et al., 2019). Moreover, in stereotypical domains such as math and reading, the relations of intrinsic value to their competence belief were stronger than the relations of a combined usefulness–importance variable to competence beliefs among children (Wigfield and Eccles, 2020). Therefore, in this study, we focus on gender differences in intrinsic value in digital learning context. Moreover, Eccles-Parsons et al. (1983) posited that individuals' competence value-related beliefs are the most proximal psychological determinants of engagement in the chosen activities. Specifically, when children place high intrinsic value on an activity, they often become deeply engaged in it and can persist at it for a long time (Eccles, 2005). As concentrating and staying focused on a learning activity in digital learning setting during COVID might be particularly challenging due to the lack of the motivational regulation in digital learning setting (Fryer and Bovee, 2016), especially for groups that might be at risk in virtual classroom, we decided to test gender differences in this component of digital learning as well. In addition, perceived teacher support is included as a contextual factor important for learning.

The first research objective focuses on differences between boys and girls in these four components of digital learning—competence beliefs, intrinsic value, learning engagement, and perceived teacher support—while the second research objective addresses differences between adolescents with different gender role self-concept—masculine, feminine, androgynous, and undifferentiated—in those components of digital learning during pandemic-induced school closures. Interaction between two gender dimensions is also tested.

In line with studies that found clear dominance of boys over girls when it comes to competence beliefs in this domain among high school students and adolescents (Mumtaz, 2001; Broos and Roe, 2006; Meelissen and Drent, 2007), we expect higher competence beliefs among boys compared to girls. Regarding the intrinsic value of digital learning, there was no directed hypothesis posed due to inconsistent results yielded from previous studies on sex differences in values toward ICT and digital learning (e.g., Ong and Lai, 2006; Price, 2006; Cuadrado-García et al., 2010; Lu and Chiou, 2010). Based on studies reporting higher engagement with computers in education purposes in boys (e.g., Drabowicz, 2014), we expect higher learning engagement in boys compared to girls in digital learning setting during COVID. As ICTs are still a gender-stereotyped domain and perceived as a masculine field, we expect students who ascribe masculine characteristics to themselves to a high degree (masculine and androgynous individuals) to show the highest levels of both competence and intrinsic value beliefs as well as engagement within the digital learning context.

Regarding the perceived teacher support, aligning with the previous study on perceived teacher support (Vekiri, 2012), we expect insignificant differences between boys and girls in perceived teacher support during pandemic-induced digital learning. However, as the orientation toward social support and social relationships is a stereotypically feminine quality, we expected the highest levels of perceived teacher support in feminine and androgynous students. For undifferentiated adolescents, the lowest levels of competence beliefs, perceived values, engagement, and perceived teacher support during digital learning are expected compared to the other three types. As competence and value-related beliefs show a decline through school years (Jacobs et al., 2002; Cimpian, 2017), as well as learning engagement (Fredricks et al., 2004) and perception of social support (Ryan et al., 1994), we controlled for age in all analyses.

## Method

### Participants, Procedure, and Context of Data Collection

The data was collected in April 2020 in Vienna, Austria, as part of a larger project investigating learning under the conditions of the COVID-19. For the purposes of this study, a subsample consisting of boys and girls only was selected, excluding 0.6% of students that declared their gender as diversed. In total, the selected study sample comprised 19,190 secondary school students (61.9% girls, *M*_age_ = 14.55, SD_age_ = 2.49, age range 10–21) from all types of Austrian secondary schools (general secondary school, technical and vocational secondary schools, and apprenticeship). Data was collected with online questionnaires. To recruit participants, we distributed the link to the online questionnaire by contacting manifold stakeholders such as school boards, educational networks, and school principals with the help of the Austrian Federal Ministry for Education, Science, and Research. Participation was voluntarily and anonymous. Only students who gave active consent were included in the dataset. In Austria, schools stopped providing onsite learning on March 16. Throughout the entire data collection period, schools were obliged to ensure that education continued in the form of digital learning. Teachers and schools were given autonomy in the organization and design of remote instruction. While there was no on-site teaching, schools remained open to provide childcare to individual students where necessary (Federal Ministry of Education, 2020b). However, this option was taken up by ~2% of the student population only (Federal Ministry of Education, 2020a).

### Measures

Due to the novelty of the COVID-19 situation, it was necessary to adapt existing scales or develop new items for scales that served as dependent variables in order to address the current circumstances. To ensure the content validity of the adapted or newly formulated items, we revised them based on expert judgments. The measures were then piloted with cognitive testing among adolescents of different ages. For details on the measures and the complete set of items, see Schober et al. (2020). All items were rated on a five-point scale ranging from 1 (strongly agree) to 5 (strongly disagree). Participants were instructed to answer the items with respect to their current digital learning activities. Analyses were conducted with recoded items so that higher values reflected higher agreement with the statements.

#### Competence Beliefs

To assess competence beliefs in digital learning, three newly developed items were used (sample item: “Overall, I am managing e-learning pretty well”), α = 0.711.

#### Intrinsic Value

Intrinsic value was assessed with three items adapted from the Scales for the Measurement of Motivational Regulation for Learning in University Students (SMR-LS; Thomas et al., 2018; sample item: “Currently, I really enjoy studying and working for school”), α = 0.916.

#### Learning Engagement

Learning engagement was measured with three slightly adapted items from the engagement subscale of the EPOCH Measure (Kern et al., 2016; sample item: “Currently when I am working on my schoolwork, I get completely absorbed in what I am doing”), α = 0.732.

#### Perceived Teacher Support

To measure the social component of digital learning, three additional items concerning interaction with teachers were used (sample item: “Currently, my teachers help me with e-learning”), α = 0.745.

#### Gender Role Self-Concept

To assess self-perceived femininity and masculinity, positive traits from the Inventory for Measuring Adolescents' Gender Role Self-Concept (GRI-JUG) were used (Krahé et al., 2007). Participants were presented with five masculine attributes (humorous, courageous, sporty, companionable, and strong; α = 0.676) and five feminine attributes (emotional, romantic, industrious, sympathetic, and empathic; α = 0.651) and were asked to rate to what extent each attribute is characteristic of them. Separate scores were calculated for masculinity and femininity. The median split procedure adopted by Spence et al. (1975) and Bem (1977) was used to determine the four types of gender role self-concepts. Participants were classified into a 2 × 2 table according to whether they fell above or below the median score on the masculinity and femininity scales. Scores falling exactly on the median were classified as “high” scores (Carver et al., 2013). In the present sample, the median masculinity score was 4.2 and the median femininity score was 4.0.

## Results

**Figure 1:**
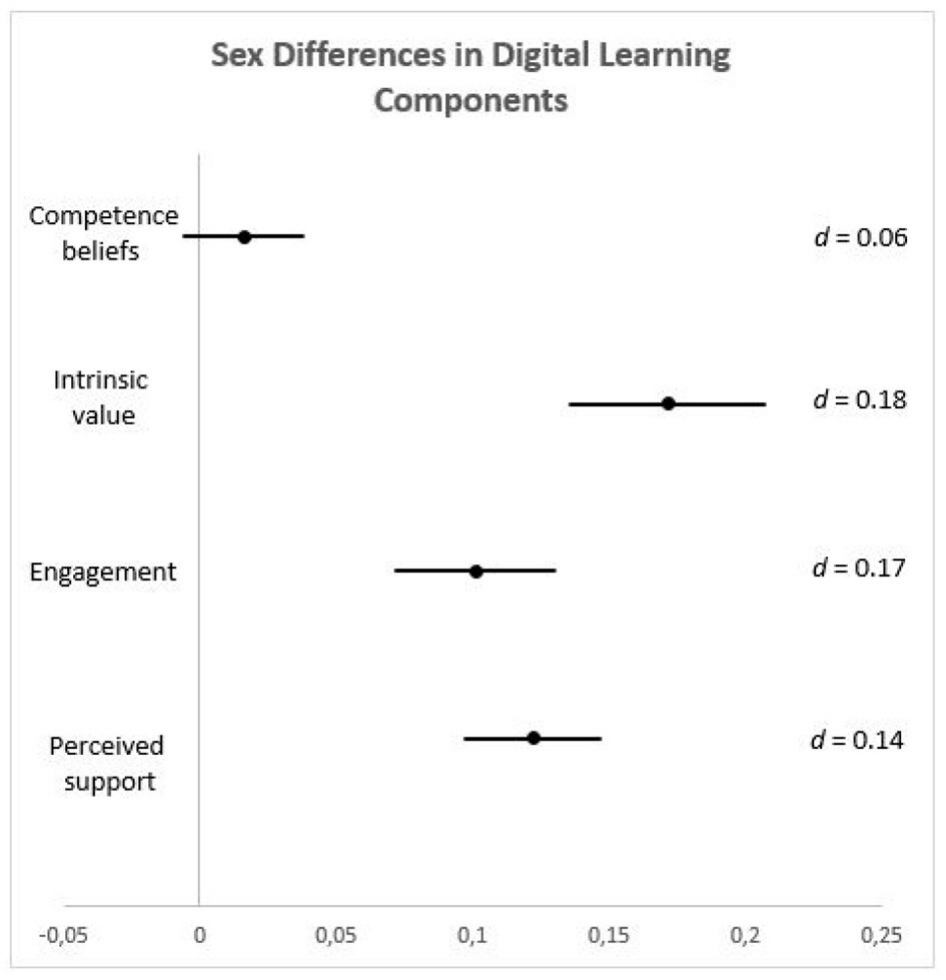
Mean differences and two-sided 95% confidence intervals for boys and girls in four digital learning components. The x-axis shows the confidence interval span. The point estimate is the mean difference between boys and girls in observed digital learning components. Cohen's d indicates effect size for the comparison between two means. Positive values indicate advantage of girls over boys in observed digital learning components.

**Figure 2:**
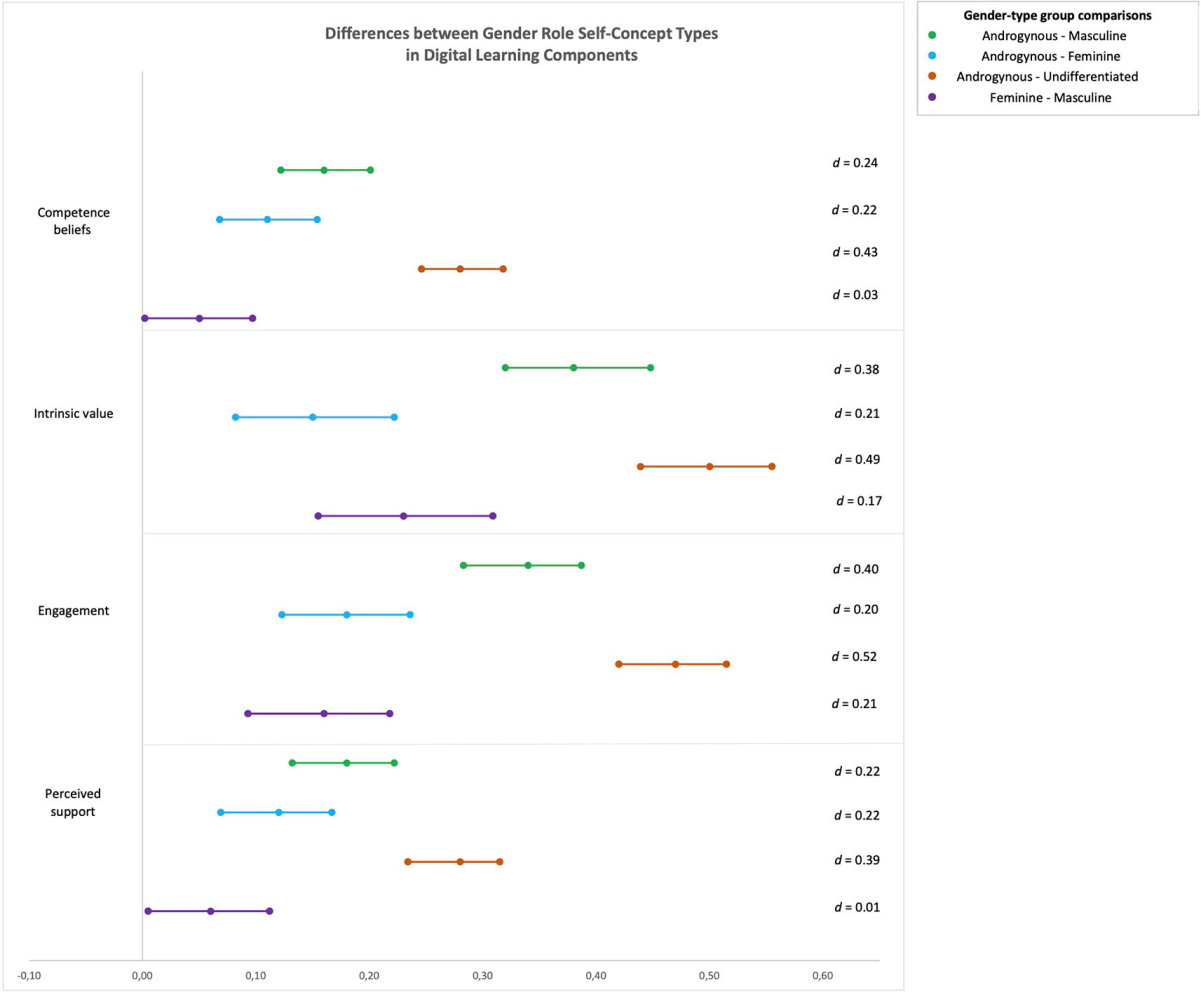
Mean differences and two-sided 95% confidence intervals for the gender role self-concept group comparisons in four digital learning components. The x-axis shows the confidence interval span. The point estimate is the mean difference between gender role self-concept types (gender-type groups) in observed digital learning components. Cohen's d indicates effect size for the comparison between two means. Positive values indicate advantage of androgynous over masculine, feminine, and undifferentiated adolescents in observed digital learning components, and advantage of feminine over masculine adolescents in observed digital learning components.

In order to examine differences in digital learning components among adolescents, four separate analyses of covariance (ANCOVAs) were conducted with sex (male/female) and gender role self-concept (androgynous/masculine/feminine/undifferentiated) as between-subject factors and age as a covariate. The mean scores for digital learning competence beliefs, intrinsic value, engagement, and perceived support in digital learning served as the dependent variables. Higher values reflect higher scores on these observed digital learning components. When interpreting the results, we focused on the effect sizes of the group differences alongside statistical significance, following Cohen's (1988) recommendations, with values around 0.10 representing small effects, values around 0.30 representing medium effects, and values > 0.50 representing large effects. Means and standard deviations for all dependent variables by sex and gender role self-concept are presented in Table 1. Effect sizes and confidence intervals for main effects of gender dimensions on four digital learning components are presented in Figure 1 (biological sex) and Figure 2 (gender role self-concept).

**Table 1:** Means and standard deviations of digital learning components by gender role self-concept and sex.

**Gender role self-concept**	**Sex**		**Competence beliefs**		**Intrinsic value**		**Learning engagement**		**Perceived teacher support**	
		***N***	***M***	**SD**	***M***	**SD**	***M***	**SD**	***M***	**SD**
Androgynous	Boys	2,175	4.22	0.751	2.95	1.184	3.40	0.945	4.20	0.841
	Girls	3,878	4.29	0.648	3.21	1.149	3.53	0.890	4.32	0.734
Masculine	Boys	2,089	4.11	0.713	2.59	1.074	3.06	0.920	4.06	0.843
	Girls	1,486	4.10	0.697	2.84	1.153	3.20	0.919	4.15	0.797
Feminine	Boys	825	4.13	0.706	2.88	1.129	3.27	0.960	4.04	0.835
	Girls	3,771	4.12	0.661	2.88	1.133	3.31	0.885	4.12	0.765
Undifferentiated	Boys	2,217	3.98	0.724	2.52	1.074	2.95	0.908	3.92	0.850
	Girls	2,733	3.96	0.704	2.61	1.109	3.05	0.906	4.01	0.788

### Competence Beliefs in Digital Learning

The results showed a statistically significant effect of age, *F*_(1, 19157)_ = 67.75, *p* = 0.000, η^2^_*p*_ = 0.004, indicating a negative relationship between age and competence beliefs in digital learning, *r*_(19, 181)_ = −0.063, *p* = 0.000. The main effect of sex was not significant, *F*_(1, 19157)_ = 2.06, *p* = 0.151, η^2^_*p*_ = 0.000. There was a statistically significant main effect of gender role self-concept after controlling for adolescents' age, *F*_(3, 19157)_ = 147.07, *p* = 0.000, η^2^_*p*_ = 0.023. A Bonferroni *post hoc* test showed that androgynous adolescents reported significantly higher competence beliefs in digital learning than masculine, feminine, and undifferentiated adolescents. Feminine adolescents exhibited a slightly higher level of competence beliefs in digital learning compared to masculine adolescents. Undifferentiated adolescents had statistically significantly lower levels of competence beliefs in digital learning than adolescents with other gender role self-concepts. The interaction between gender role self-concept and sex was statistically significant, *F*_(3, 19157)_ = 5.60, *p* = 0.001, η^2^_*p*_ = 0.001, indicating higher levels of competence beliefs in digital learning among androgynous girls compared to androgynous boys and all other groups. Feminine boys and girls, masculine boys and girls, and undifferentiated boys and girls achieved similar scores.

### Intrinsic Value

The results showed a statistically significant effect of age, *F*_(1, 19157)_ = 209.64, *p* = 0.000, η^2^_*p*_ = 0.011, indicating a negative relationship between age and intrinsic value, *r*_(19, 179)_ = −0.094, *p* = 0.000. The main effect of sex was statistically significant after controlling for adolescents' age, *F*_(1, 19157)_ = 89.50, *p* = 0.000, η^2^_*p*_ = 0.005, with girls reporting higher intrinsic value of digital learning compared to boys. There was also a statistically significant main effect of gender role self-concept after controlling for adolescents' age, *F*_(3, 19157)_ = 194.91, *p* = 0.000, $\eta_{p}^{2}$ = 0.030. A Bonferroni *post hoc* test showed that androgynous adolescents reported significantly higher intrinsic value than masculine, feminine, and undifferentiated adolescents. Feminine adolescents showed higher levels of intrinsic value compared to masculine adolescents. Undifferentiated adolescents had statistically significantly lower intrinsic value than adolescents with other gender role self-concepts. The interaction between gender role self-concept and sex was also statistically significant, *F*_(3, 19157)_ = 12.04, *p* = 0.000, η^2^_*p*_ = 0.002, indicating higher levels of intrinsic value in androgynous girls compared to androgynous boys and all other groups, as well as in masculine girls compared to masculine boys. Feminine girls and boys achieved similar results, as did undifferentiated boys and girls.

### Learning Engagement

The results showed a statistically non-significant effect of age, *F*_(1, 19157)_ = 0.46, *p* = 0.500, η^2^_*p*_ = 0.000. The main effect of sex was statistically significant, *F*_(1, 19157)_ = 47.21, *p* = 0.000, η^2^_*p*_ = 0.002, with girls scoring higher on learning engagement compared to boys. The main effect of gender role self-concept after controlling for adolescents' age was also statistically significant, *F*_(3, 19157)_ = 247.44, *p* = 0.000, η^2^_*p*_ = 0.037. A Bonferroni *post hoc* test showed that androgynous adolescents reported statistically significantly higher levels of learning engagement than masculine, feminine, and undifferentiated adolescents. Feminine adolescents showed a higher level of learning engagement compared to masculine adolescents. Undifferentiated adolescents had lower levels of learning engagement than adolescents with other gender role self-concepts. The interaction between gender role self-concept and sex was not statistically significant, *F*_(3, 19157)_ = 1.72, *p* = 0.161, η^2^_*p*_ = 0.000.

### Perceived Teacher Support in Digital Learning

The results showed a statistically significant effect of age, *F*_(1, 19157)_ = 602.61, *p* = 0.000, η^2^_*p*_ = 0.030, indicating a negative relationship between age and perceived teacher support in digital learning, *r*_(19, 181)_ = −0.170, *p* = 0.000. The main effect of sex was also statistically significant after controlling for adolescents' age, *F*_(1, 19157)_ = 92.47, *p* = 0.000, η^2^_*p*_ = 0.005, with girls reporting higher perceived teacher support in digital learning compared to boys. There was also a statistically significant main effect of gender role self-concept after controlling for adolescents' age, *F*_(3, 19157)_ = 110.70, *p* = 0.000, η^2^_*p*_ = 0.017. A Bonferroni *post hoc* test showed that androgynous adolescents reported statistically significantly higher levels of perceived teacher support in digital learning than masculine, feminine, and undifferentiated adolescents. Feminine adolescents reported higher perceived teacher support in digital learning compared to masculine adolescents. Undifferentiated adolescents reported statistically significantly lower perceived teacher support in digital learning than adolescents with other gender role self-concepts. The interaction between gender role self-concept and sex was not statistically significant, *F*_(3, 19157)_ = 0.76, *p* = 0.515.

## Discussion

The main goal of this study was to investigate the gender differences in a digital learning context during a period of pandemic-induced school closures, including both biological sex and gender role self-concept in tackling the differences in this stereotyped domain. The study encompassed four components of digital learning identified as not only important for learning success but also susceptible for stereotyped gender gap: competence beliefs, intrinsic value, engagement, and perceived teacher support. The first objective of the study focused on sex differences in the examined components of digital learning.

Our results showed no differences between boys and girls in competence beliefs in digital learning, indicating that girls and boys had equal levels of perceived abilities in digital learning. Although previous studies have revealed higher levels of competence beliefs related to computers and technologies in general among adolescent boys (see Vekiri and Chronaki, 2008), our results showed equality between boys and girls with respect to managing digital learning, using technologies and technical equipment to complete their school tasks and comprehension of tasks performed in a digital learning format. This finding is inconsistent with our assumption, but it can be explained with girls' general higher academic competence beliefs in adolescence (Britner and Pajares, 2001), which potentially translated into digital learning setting leveling thus the sex differences in this context. On the other hand, while studies have continuously showed higher engagement with computers in general among boys (Colley and Comber, 2003; Drabowicz, 2014), girls exhibited higher digital learning engagement in our study. This is not surprising given girls' higher levels of engagement in school-related tasks in general (Lam et al., 2012). It has been shown that even though boys are perceived as more skilled than girls (Bian et al., 2017), girls are more engaged with learning activities and more study oriented (Van Houtte, 2004). Thus, it might be that girls transferred their established learning practices into new learning context when schools switched to digital learning. This seems especially plausible given the unpreparedness of schools and teachers for this new teaching context (Hodges et al., 2020), which could have caused them to apply usual didactic techniques from face-to-face teaching, without fully adapting to the digital context. Thus, stereotypical aspects of digital context potentially were not pronounced enough to threaten girls' engagement. In differently organized digital learning setting where typically masculine technical skills would be more required, results might show stereotypical results of boys' dominance in engagement with technology even in a learning context.

Our results showed the same non-stereotypical pattern for intrinsic value of digital learning, which corresponds to the results of some previous studies finding higher intrinsic motivation in digital learning contexts among girls (e.g., McSporran and Young, 2001; Price, 2006). Various studies have shown that boys are less motivated than girls and have less positive attitudes toward school in general (Cox, 2000; Francis, 2000), which potentially overflowed into digital learning context during this pandemic. This is especially plausible given that data was collected soon after schools in Austria switched to online learning. Results might be different now after a year of digital learning practice when both boys and girls are more habituated to it.

Regarding the contextual factor in learning, our results have shown higher perceived teacher support among girls than among boys. While previous studies on ICT did not find differences between boys and girls in perceived teacher expectations and support (Vekiri, 2010), this finding is not surprising given the stronger orientation toward social relationships and social support in the feminine gender role associated with girls compared to the masculine gender role associated with boys in Western societies (Helgeson, 1994; Korlat et al., 2021). In line with gender stereotypes, previous studies showed that girls rely more upon social support, especially in difficult or stressful situations (Helsen et al., 2000; Tamres et al., 2002), which might have been the case for digital learning during COVID-19. In addition, it has been shown that girls value student–teacher interaction more than boys do (e.g., Frymier and Houser, 2000). Thus, girls might be more proactive than boys in reaching out to teachers, thus establishing better relationships with them. On the other hand, teachers might provide more support to girls due to stereotypes about ICT and girls' potential disadvantages in the virtual classroom. Learning heavily relies on interactions between students and teachers (Taylor et al., 2007), so the potentially lower social support perceived by boys could affect their learning processes, particularly in light of the fact that the digital context in pandemic-induced learning might require more active interaction with the teacher than in-person instruction.

Taken together, our results challenge the notion of girls' potential disadvantages in the virtual classroom and reveal their relatively higher levels of perceived social support from teachers, intrinsic value, and engagement for digital learning. This calls attention to the challenges boys might face in the digital learning context, which could potentially intensify boys' existing underperformance in terms of overall academic achievement (Duckworth and Seligman, 2006; Hartley and Sutton, 2013). It is important to note that the effect sizes of the sex differences found in our study are small (Cohen's *d* ranging from 0.14 to 0.18), supporting the gender similarity hypothesis (Hyde, 2005), according to which gender differences on most psychological variables are small or close to zero. Nevertheless, boys' potential disadvantages regarding interaction with teachers, intrinsic value, and learning engagement during the pandemic-induced period of mandatory digital learning should not be easily discarded. Given the possibility that learning during pandemic might be organized in a way that resembles face-to-face learning—where boys lack engagement and study-oriented culture (Van Houtte, 2004)—but in a distance form when students are forced to organize their learning autonomously without external regulation as in face-to-face learning (Huber et al., 2020), schooling during COVID could create an even higher risk for boys' academic achievement compared to pre-pandemic conditions. Moreover, boys' higher engagement with computers for entertainment purposes such as video games (Terlecki et al., 2011; Drabowicz, 2014) might have a negative influence for their self-regulated learning, posing great challenge to their focus and learning process in this context. Hence, schools and teachers should take into account all potential threats to both boys' and girls' learning process when organizing teaching in digital context during pandemic.

As ICTs are still a gender-stereotyped domain and perceived as a masculine field, it could be that gender differences in digital learning map onto students' gender role self-concept rather than their biological sex. Thus, the second objective of our study was to investigate differences between boys and girls with different gender role self-concepts in the studied components of digital learning during the pandemic-induced school closures. As expected, feminine adolescents reported higher levels of perceived social support than masculine and undifferentiated adolescents. This finding supports the notion of the compatibility between gender roles and gendered activities proposed by Eccles-Parsons et al. (1983) and Eccles (2009), as social support and social relationships represent the core of stereotypical femininity. Surprisingly, and contrary to our expectations, femininity was a contributing factor to higher levels of *stereotypically masculine* components of digital learning as well: feminine students exhibited higher levels of competence beliefs, intrinsic value, and engagement in digital learning compared to masculine and undifferentiated students. One explanation for this could be the higher relevance of femininity compared to masculinity for adolescents in the school context. Studies have found stronger school-related self-esteem and stronger feelings of belonging at school among feminine adolescents (Skinner et al., 2019). Moreover, feminine students are often more liked by teachers (Heyder and Kessels, 2013), which, alongside higher perceived teacher support, could contribute to higher intrinsic value and engagement in digital learning in girls, even in the digital context. However, the effect sizes for the differences between adolescents with feminine and masculine gender role self-concepts on all variables were small or close to zero (Cohen's *d* ranging from 0.01 for perceived support to 0.21 for learning engagement). Importantly, our results showed clear advantages of androgyny over both femininity and masculinity for digital learning with medium to large effect sizes, indicating the higher value of possessing both feminine and masculine characteristics than one sort only. Although Eccles (2009) assumed educational benefits in case of fitness between the stereotypicality of a task and one's gender role self-concept, this finding is not surprising given the broader repertoire of traits and behaviors (e.g., Pauletti et al., 2017) in androgynous individuals compared to others. This finding confirms better coping in different life situations related to androgyny suggested by Bem (1981), applied to altered learning setting in a pandemic era. Interestingly, it seems that girls with androgynous characteristics have the clearest advantage over boys and girls with different gender role self-concept in competence and value-related beliefs regarding digital learning. As both ability and value beliefs are important for learning achievement (Wigfield and Eccles, 2000), androgynous girls might benefit the most from the pandemic-induced digital learning situation. Similarly, masculine girls showed higher levels of intrinsic value in the digital learning context compared to masculine boys. This is in line with the general advantage of girls over boys found in this study, however only with the small magnitude. As expected, undifferentiated adolescents achieved lower scores in all digital learning components under study, due to a lack of beneficial attributes and behaviors for coping.

In line with previous studies showing a decline in competence and value-related beliefs throughout adolescence (Jacobs et al., 2002; Cimpian, 2017), our results showed lower competence beliefs and intrinsic value for digital learning with increasing age. One reason for that might be an over-optimistic assessment in young children about their competencies in different areas and consequent high placed value (see Cimpian, 2017 for discussion). In addition, scholars argue that learning becomes more and more decontextualized and performance-oriented in adolescence, which undermines intrinsic motivation (Gnambs and Hanfstingl, 2016). At the same time, adolescence is a period where social relations and peers increase in importance (Simons-Morton and Chen, 2009; LaFontana and Cillessen, 2010), which might take adolescents' focus off learning. Accordingly, younger students were found to report higher perceived teacher support in this study. While the effect sizes are very small, it could be that teachers provide more assistance to younger students in digital learning, taking into account their lower experience with ICT and potentially longer adaptation period to this new learning setting. As the results indicate older students, along with boys, might particularly struggle with digital learning, teachers and schools should offer more support to them and pay particular attention to their management of school-related tasks in this new learning context. In addition, developing curricular activities and a virtual classroom environment that enhance both feminine and masculine traits and behaviors in both boys and girls may enhance their digital learning in the COVID-19 era.

### Limitations and Future Directions

While this study has several strengths, including a large sample size, some limitations must be considered. First, even though pandemic-induced school closure provides a good opportunity to investigate digital learning in a large sample of students, schools employed different digital platforms and teaching methods (e.g., synchronous and asynchronous) to support their teaching during the school closures. Future studies should investigate the role of gender in terms of both biological sex and gender role self-concept in digital learning settings with more uniform teaching and learning practices. Second, the data was collected online, which led to a self-selected sample. Third, future studies should include other value components (utility, importance, and cost) of the expectancy-value model, other contextual variables such as parental beliefs, and variables regarding the digital learning environment such as access to ICT. Finally, this study only takes into account positive aspects of gender role self-concept. Future studies should include both positive and negative aspects in order to more fully investigate the role of gender and stereotyped components of digital learning.

## Data Availability Statement

The datasets presented in this study can be found in online repositories. The names of the repository/repositories and accession number(s) can be found at: https://doi.org/10.11587/VRQL3B.

## Ethics Statement

Ethical review and approval was not required for the study on human participants in accordance with the local legislation and institutional requirements. Written informed consent for participation was not provided by the participants' legal guardians/next of kin because data was collected online due to circumstances of the COVID-19 and only consent from students was collected. Only students who gave active consent were included in the data set.

## Author Contributions

All authors listed have made a substantial, direct and intellectual contribution to the work, and approved it for publication.

## Conflict of Interest

The authors declare that the research was conducted in the absence of any commercial or financial relationships that could be construed as a potential conflict of interest.
